# A Genome Wide Association Study Links Glutamate Receptor Pathway to Sporadic Creutzfeldt-Jakob Disease Risk

**DOI:** 10.1371/journal.pone.0123654

**Published:** 2015-04-28

**Authors:** Pascual Sanchez-Juan, Matthew T. Bishop, Gabor G. Kovacs, Miguel Calero, Yurii S. Aulchenko, Anna Ladogana, Alison Boyd, Victoria Lewis, Claudia Ponto, Olga Calero, Anna Poleggi, Ángel Carracedo, Sven J. van der Lee, Thomas Ströbel, Fernando Rivadeneira, Albert Hofman, Stéphane Haïk, Onofre Combarros, José Berciano, Andre G. Uitterlinden, Steven J. Collins, Herbert Budka, Jean-Philippe Brandel, Jean Louis Laplanche, Maurizio Pocchiari, Inga Zerr, Richard S. G. Knight, Robert G. Will, Cornelia M. van Duijn

**Affiliations:** 1 Neurology Department, University Hospital “Marqués de Valdecilla”. Instituto de Investigación “Marqués de Valdecilla” IDIVAL and Centro de Investigación Biomédica en Red sobre Enfermedades Neurodegenerativas (CIBERNED). Santander, Spain; 2 The National Creutzfeldt-Jakob disease Research and Surveillance Unit, University of Edinburgh, United Kingdom; 3 Institute of Neurology, Medical University Vienna, Vienna, Austria; 4 Chronic Disease Programme and CIBERNED. Carlos III Institute of Health. Madrid. Spain; 5 Alzheimer Disease Research Unit, CIEN Foundation, Carlos III Institute of Health, Alzheimer Center Reina Sofia Foundation, Madrid, Spain; 6 Department of Epidemiology, Erasmus Medical Centre, Rotterdam, the Netherlands; 7 Institute of Cytology and Genetics SB RAS, Novosibirsk, Russia; 8 Novosibirsk State University, Novosibirsk, Russia; 9 Department of Cell Biology and Neurosciences Instituto Superiore di Sanità, Roma, Italy; 10 Department of Pathology, The University of Melbourne, Parkville, 3010, Australia; 11 Department of Neurology, Clinical Dementia Centre, University Medical Center and German Center for Neurodegenerative Diseases (DZNE)—site Göttingen, Göttingen, Germany; 12 Fundación Pública Galega de Medicina Xenómica, CIBERER, Grupo de Medicina Xenómica-Universidad de Santiago de Compostela, Santiago de Compostela, Spain; 13 Center of Excellence in Genomic Medicine Research (CEGMR), King Abdulaziz University, Jeddah, KSA; 14 Department of Internal Medicine, Erasmus Medical Center, Rotterdam, the Netherlands; 15 Sorbonne Universités, UPMC Univ Paris 06 UMR S 1127, and Inserm, U 1127, and CNRS UMR 7225, and ICM, F-75013, Paris, France; AP-HP, Hôpital de la Pitié Salpêtrière, Cellule Nationale de Référence des maladies de Creutzfeldt-Jakob, F-75013, Paris, France; 16 Service de biochimie et biologie moleculaire, Laboratoire associé au CNR "ATNC", Hôpital Lariboisiére, AP-HP, Paris, France; University of Verona, ITALY

## Abstract

We performed a genome-wide association (GWA) study in 434 sporadic Creutzfeldt-Jakob disease (sCJD) patients and 1939 controls from the United Kingdom, Germany and The Netherlands. The findings were replicated in an independent sample of 1109 sCJD and 2264 controls provided by a multinational consortium. From the initial GWA analysis we selected 23 SNPs for further genotyping in 1109 sCJD cases from seven different countries. Five SNPs were significantly associated with sCJD after correction for multiple testing. Subsequently these five SNPs were genotyped in 2264 controls. The pooled analysis, including 1543 sCJD cases and 4203 controls, yielded two genome wide significant results: rs6107516 (p-value=7.62x10^-9^) a variant tagging the prion protein gene (*PRNP*); and rs6951643 (p-value=1.66x10^-8^) tagging the Glutamate Receptor Metabotropic 8 gene (*GRM8)*. Next we analysed the data stratifying by country of origin combining samples from the pooled analysis with genotypes from the *1000 Genomes Project* and imputed genotypes from the *Rotterdam Study* (Total n=12967). The meta-analysis of the results showed that rs6107516 (p-value=3.00x10^-8^) and rs6951643 (p-value=3.91x10^-5^) remained as the two most significantly associated SNPs. Rs6951643 is located in an intronic region of *GRM8*, a gene that was additionally tagged by a cluster of 12 SNPs within our top100 ranked results. *GRM8* encodes for mGluR8, a protein which belongs to the metabotropic glutamate receptor family, recently shown to be involved in the transduction of cellular signals triggered by the prion protein. Pathway enrichment analyses performed with both Ingenuity Pathway Analysis and ALIGATOR postulates glutamate receptor signalling as one of the main pathways associated with sCJD. In summary, we have detected GRM8 as a novel, non-PRNP, genome-wide significant marker associated with heightened disease risk, providing additional evidence supporting a role of glutamate receptors in sCJD pathogenesis.

## Introduction

Sporadic Creutzfeldt-Jakob disease (sCJD), although rare, with a yearly incidence of one to two cases per million, is the most common form of human prion disease. This group of disorders is characterized by spongiform changes in the brain, as well as accumulation of misfolded, often protease-resistant, conformers (PrP^Sc^) of the normal prion protein (PrP^C^). The PrP^C^ gene (*PRNP*) plays a central role in prion disease susceptibility. Expression of PrP^C^ is indispensable for disease transmission [1] and the polymorphism coding for methionine (M) or valine (V) at codon 129 (*PRNP* M129V) has been linked to disease risk [2]. Homozygosity at *PRNP* M129V has been consistently associated to sCJD, being one of the strongest common genetic risk factors reported for neurodegenerative diseases. The remarkable disease-determining effect of this *PRNP* polymorphism is observed in variant CJD, a subtype acquired from dietary exposure to bovine spongiform encephalopathy [3], where all definite and probable clinical cases studied to date have been *PRNP*129MM [4].

Similar to other diseases, several genetic association studies with candidate genes have been performed on sCJD susceptibility [5]. Only one previous genome wide association study (GWAS) of sCJD risk has been published to date, showing that the *PRNP* locus was strongly associated with disease risk, specifically with rs1799990 (*PRNP* M129V) [6].

To further scrutinize genomic variations related to sCJD risk we have performed a three-stage GWAS encompassing a total of 1,543 sCJD cases and 4,203 controls, as well as a meta-analysis encompassing data from the *1000 Genomes Project* and imputations from the *Rotterdam Study*.

## Results

Demographic and clinical features of the sCJD case populations are shown in S1 Table. The Q-Q plots for autosomal and X chromosome SNPs are given in S1 Fig; during discovery (stage one) the genomic inflation factor λ was 1.053 for autosomal and 1.057 for X chromosome SNPs.

S2 Table shows the top 100 SNPs associated with sCJD at discovery stage, sorted by allelic differences p-value. A total of 23 SNPs were taken forward to replication. In stage two, we successfully genotyped 22 of the 23 SNPs with the Sequenom iPLEx GOLD platform in an independent population of 1,109 samples of sCJD. Table 1 shows that five SNPs remained significant after Bonferroni correction. In stage three we genotyped those five SNPs in a population of 2,264 independent controls with Sequenom iPLEx GOLD. S2 Fig depicts neatly separated genotype clusters of the five SNPs studied in stage three. Only two SNPs were successfully replicated at stage three and reached genome wide significant p-values after meta-analysis of discovery and replication results: rs6107516 (p-value = 7.62x10^-9^) tagging *PRNP* and in linkage disequilibrium (LD) with *PRNP* M129V (rs1799990), and rs6951643 (p-value = 1.66x10^-8^) an intronic SNP within *GRM8* (Glutamate Receptor Metabotropic 8) in chromosome 7. Table 2 shows differences in allelic frequencies in the discovery, replication and pooled population sets including 1,543 sCJD cases and 4,203 controls. The A allele of rs6951643 was associated with a 1.27 fold increased risk of sCJD (95%CI = 1.17–1.38), and this effect was consistently observed across all tested populations (Table 3).

**Table 1:** SNPs genotyped in stage two.

			Stage one: Discovery			Stage two: Replication in independent sCJD Cases	
SNP	CJD risk allele in Discovery population	GENE tagged	Controls Frequencies of sCJD risk allele (n = 1939)	Cases Frequencies of sCJD risk allele (n = 434)	P1df*	Cases Frequencies of sCJD risk allele (n = 1109)	P-value**
**rs10061929**	A	FLJ43080	0.159	0.224	4.58E-05	0.200	5.89E-05
rs10915708	T	NA	0.140	0.207	1.35E-06	0.158	0.0626
rs11075924	C	NA	0.498	0.565	5.52E-05	0.500	0.8808
rs11245373	T	NA	0.037	0.069	2.48E-05	0.049	0.0238
rs12102156	T	NA	0.863	0.912	2.00E-05	0.849	0.1457
rs12188818	C	NA	0.796	0.859	6.44E-05	0.809	0.2544
rs12419710	A	NA	0.210	0.279	2.52E-05	0.219	0.3746
rs17060736	G	NA	0.112	0.160	7.13E-05	0.121	0.3134
**rs17115017**	A	GRIA1	0.050	0.082	2.23E-04	0.072	5.00E-04
rs17833759	C	GRIN2B	0.085	0.140	7.74E-07	0.095	0.1955
rs196940	C	ERN1	0.758	0.818	6.19E-05	0.758	0.9631
rs2240344	G	NA	0.432	0.600	4.47E-19	0.443	0.4194
rs2627829	A	INPP4B	0.939	0.970	1.83E-04	0.931	0.2188
rs392184	T	MACROD2	0.052	0.096	2.00E-06	0.07	0.003
rs565559	T	NA	0.553	0.618	2.02E-04	0.579	0.057
rs6027482	C	LOC100131710	0.187	0.247	6.69E-05	0.181	0.5476
**rs6107516**	C	PRNP	0.752	0.827	6.92E-06	0.804	3.27E-06
rs6463269	G	NA	0.084	0.134	3.53E-06	GENOTYPE FAILED	-
rs6496239	T	NA	0.805	0.862	4.96E-05	0.821	0.1195
rs6820644	T	NA	0.357	0.433	3.40E-06	0.647	0.7566
**rs6951643**	A	GRM8	0.507	0.592	8.09E-06	0.573	6.79E-07
**rs9521699**	A	COL4A2	0.137	0.194	3.84E-05	0.173	2.00E-04
rs9830696	T	NA	0.120	0.173	1.02E-05	0.104	0.0711

sCJD: Sporadic Creutzfeldt-Jakob Disease

In bold SNPs significant after Bonferroni correction (Replication P-value <0.0023)

* P-value for allellic diferences adjusted by Country of origin by PCA

**Chi Squared p-value from comparison between Allele frequencies of independent sCJD cases and stage one controls

NA: SNP in intergenic region.

**Table 2:** SNPs studied in stage three.

			Discovery Population				Replication Population			Pooled Samples		
ID	CJD risk allele in Discovery population	GENE tagged	Case, Control Frequencies of CJD risk allele (n = 434, n = 1939)	P-value	P-value*	OR (95%CI)	Case, Control Frequencies of CJD risk allele (n = 1109, n = 2264)	P-value	OR (95%CI)	Case.Control Frequencies of CJD risk allele (n = 1543, n = 4203)	P-value	OR (95%CI)
rs10061929	A	FLJ43080	0.224, 0.159	4.30E-06	4.58E-05	1.53 (1.27–1.83)	0.200, 0.196	0.6882	1.03 (0.90–1.17)	0.207, 0.179	0.0007	1.20 (1.08–1.33)
rs17115017	A	GRIA1	0.082, 0.050	3.00E-04	2.23E-04	1.69 (1.27–2.24)	0.072, 0.077	0.4434	1.08 (0.89–1.31)	0.075, 0.065	0.0597	1.17 (0.99–1.37)
rs6107516	C	PRNP	0.827, 0.752	2.07E-06	6.92E-06	1.58 (1.31–1.91)	0.804, 0.766	**5.00E-04**	1.25 (1.10–1.42)	0.811, 0.759	**7.62E-09**	1.35 (1.22–1.50)
rs6951643	A	GRM8	0.592, 0.507	6.73E-06	8.09E-06	1.41 (1.21–1.64)	0.573, 0.529	**7.00E-04**	1.19 (1.08–1.32)	0.578, 0.519	**1.66E-08**	1.27 (1.17–1.38)
rs9521699	A	COL4A2	0.194, 0.137	2.41E-05	3.84E-05	1.51 (1.25–1.83)	0.173, 0.173	0.9723	1.00 (0.88–1.15)	0.179, 0.157	0.0045	1.17 (1.05–1.31)

* Adjusted by Country of origin by PCA.

**Table 3:** - rs6951643 pooled genotypes by country.

Population	GG n (%)	AG n (%)	AA n (%)	A allele n (%)	G allele n (%)
**CONTOLS**					
UK	364 (24.6)	741 (50.0)	377 (25.4)	1495 (50.4)	1469 (49.6)
NL	118 (22.4)	265 (50.3)	144 (27.3)	553 (52.5)	501 (47.5)
Spain	488 (22.3)	1097 (50.0)	608 (27.7)	2313 (52.7)	2073 (47.3)
**CASES**					
UK	28 (10.5)	155 (58.3)	83 (31.2)	321 (60.3)	211 (39.7)
NL	23 (18.1)	62 (48.8)	42 (33.1)	146 (57.5)	108 (42.5)
Germany	66 (16.7)	217 (54.8)	113 (28.5)	443 (55.9)	349 (44.1)
Italy	43 (15.0)	140 (48.8)	104 (36.2)	348 (60.6)	226 (39.4)
Australia	10 (20.8)	26 (54.2)	12 (25.0)	50 (52.1)	46 (47.9)
France	23 (15.3)	77 (51.3)	50 (33.3)	177 (59.0)	123 (41.0)
Spain	35 (17.2)	110 (54.2)	58 (28.6)	226 (55.7)	180 (44.3)
Austria	11 (20.0)	27 (49.1)	17 (30.9)	61 (55.5)	49 (44.5)
**ALL CONTOLS**	970 (23.1)	2103 (50.0)	1129 (26.9)	4361 (51.9)	4043 (48.1)
**ALL CASES**	239 (15.6)	814 (53.1)	479 (31.3)	1772 (57.8)	1292 (42.2)
**HapMap Frequencies (CEU)**	27 (23.9)	59 (52.2)	27 (23.9)	113 (0.50)	113 (0.50)

**Fig 1:**
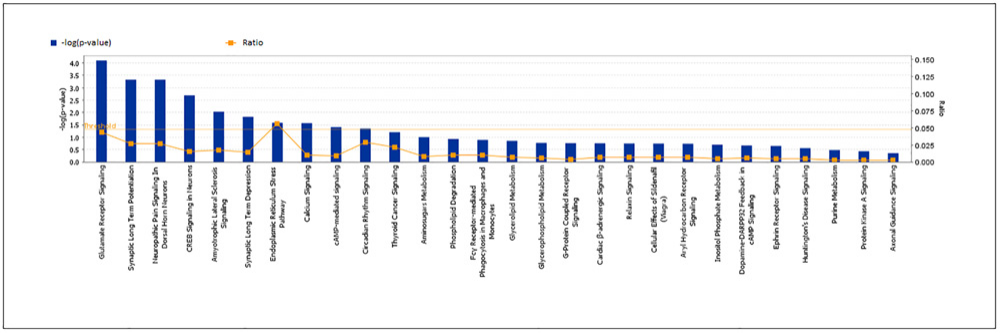
Canonical pathway analysis performed by IPA software including those genes tagged by SNPs with p-value <0.001 in our GWAs analysis. The horizontal axis represents the pathways identified. The ratio (vertical axis, right) is calculated by the numbers of genes in a given pathway that meet cutoff criteria, divided by total numbers of genes that make up that pathway. The orange line stands for the threshold above which there are statistically significantly (by default P<0.05). The vertical axis (left) shows the −log of the p-value calculated based on Fisher’s exact test.

Rs6107516 (p-value = 3.00x10^-8^) and rs6951643 (p-value = 3.91x10^-5^) remained as the two most statistically significant associations in a meta-analysis of the discovery data adjusted for PCAs and analysis of replication samples stratified by country. This meta-analysis included discovery and replication samples plus data from the *1000 Genomes Project* and imputed genotypes from the *Rotterdam Study* (Total n = 12967), (Table 4). Within the top 100 SNPs, there were 12 tagging GRM8 (S2 Table). Pathway analysis using IPA showed that glutamate receptor signalling was the most over-represented canonical pathway within our top results (p-value = 8.01x10^-5^) (Fig 1). Analysis with ALIGATOR software showed (S3 Table) that the GO category glutamate receptor activity was the 4th most represented amongst our top results. Three genes from the GO category "glutamate receptor pathway" (S2 Table) were within our top 100 SNPs (*GRM8*, *GRIN2B* and *GRIA1*). Fine mapping in search for functional variants linked to rs6951643 was attempted sequencing the 11 exons of *GRM8* gene and the corresponding intronic flanking regions in 96 sCJD patients. We found 8 intronic SNPs (rs73231278, rs62468898, rs1008274, rs111546739, rs17685327, rs6951643, rs2074012, rs35648111) and one exonic non-coding SNP (rs34182595). Functional prediction analysis of these genetic variants by FuncPred or RESCUE-ESE showed no indication of regulatory implications. In the Gtex eQTL database we found no eQTL associated to any of the *GRM8* SNPs. There is no co-expression of GRM8 and *PRNP* in blood eQTLs in Genenetwork. However, based on PITA prediction we found that rs34182595, which is an insertion/deletion SNP in exon 11 in partial LD with rs6951643 (D' = 0.41; r^2^ = 0.16), is located in a target site for microRNAs (miR-103 and miR-107), a finding that has been previously reported [7].

**Table 4:** Meta-analysis by country of origin.

MarkerName	Effect estimates of discovery analysis corrected for population substructure by PCA			Meta-analysis of effect estimates of replication analysis stratified by country of origin				Meta-analysis of discovery and replication		
	β	SE	P-value	β	SE	P-value	β Direction*	β	SE	P-value
rs6107516	0.4243	0.0944	6.92E-06	0.2327	0.0637	0.000262	₊₊₊₊₊	0.2927	0.0528	3.00E-08
rs17115017	-0.5363	0.1453	0.000223	-0.1420	0.1061	0.181	₋₋₋₊₊	-0.2792	0.0857	0.001123
rs9521699	-0.4087	0.0993	3.84E-05	-0.0233	0.0723	0.7468	₊₋₊₋₋	-0.157	0.0585	0.007242
rs6951643	-0.3472	0.0778	8.09E-06	-0.1033	0.0545	0.05821	₋₊₊₊₊	-0.1837	0.0447	3.91E-05
rs10061929	-0.3737	0.0917	4.58E-05	-0.0289	0.0701	0.6801	₋₋₋₊₊	-0.1562	0.0557	0.005048

* Concordance between discovery population βs and each of the five replication populations; PCA, Principal Component Analysis; SE, standard Error.

**Fig 2:**
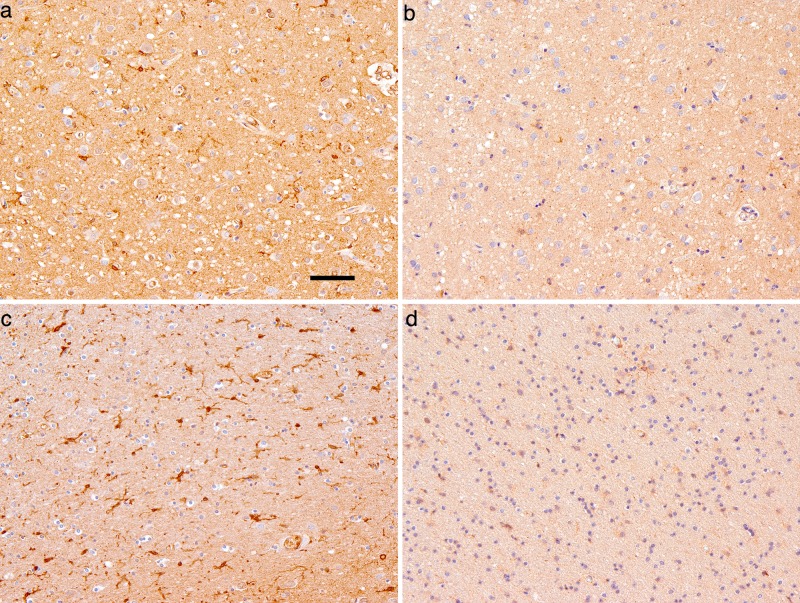
Representative photomicrographs of a sCJD case with AA (a, c) and a sCJD case with GG (b, d) rs6951643 genotype showing differences in mGluR8 immunostaining of microglial cells in the temporal cortex (a, b) and temporal white matter (c, d). Bar represents 50 μm for all images.

We then performed an immunohistochemical study in brain samples from 48 sCJD patients. Immunoreactivity for mGluR8 was observed in neurons and microglial cells (both in the white and grey matter) While in neurons we did not observe obvious differences we found a trend towards higher combined score of mGluR8 immunoreactivity in microglia related to the A allele at rs6951643 (Fig 2); however, these differences were not statistically significant (ordinal regression A-carriers versus A-non-carriers adjusting by c129 and levels of microglia in temporal cortex; p-value = 0.093). S3 Fig shows semi-quantitative assessments of mGluR8 expression in microglia (0 to 3) in the temporal region across the three rs6951643 genotypes. We found no association between rs6951643 genotypes and patient’s age of disease onset or disease duration.

## Discussion

In this study we report a non-*PRNP* genetic risk variant for sCJD, *GRM8*. Moreover, building on findings from previous studies [8], pathway analyses yielded glutamate receptor signalling as one of the main pathways linked to sCJD pathogenesis.

A previous GWAS of prion diseases employed 1,259 sCJD samples, in addition to other disease subtypes and 6,015 shared controls [6]. In the sCJD sub-group and in a meta-analysis including all prion cases only variants in *PRNP* were found to be significantly associated with disease risk. Several SNPs outside *PRNP* were identified, but in contrast to our multi-national disease cohort the association with sCJD was not homogeneous across the different geographical groups. Our main novel finding (rs6951643) was not statistically significant in that analysis. One possible explanation for this discrepancy is the fact that both studies have limited power to detect small effects due to the overall restricted case numbers included for study and therefore a SNP with a relatively modest OR of 1.27 might not be detected by all analyses. The experience and insights drawn from GWAS performed in more prevalent disorders, such as Alzheimer’s disease, shows that increasing sample size and performing meta-analysis might be essential in order to discover and confirm, relatively small-effect genetic risk factors. However, it is worth re-iterating that in our study, the association with rs6951643 was consistent across the geographically diverse sCJD population tested, showing an over-representation of the A allele in cases, and across the different genotyping methods used.

A limitation of our primary analysis was the fact that we were not able to adjust by country of origin at the replication stage. In order to overcome this caveat we performed a meta-analysis including control data from the *1000 Genomes Project* and imputed data from the Rotterdam Study. In the meta-analysis of the stratified analysis by country of origin the association of both SNPs became less significant suggesting that some signal might be caused by population stratification. Despite the fact that both variants were nominally significant in the meta-analysis of the replication data, the *PRNP* variant was the only genome wide significantly associated to sCJD in the pooled meta-analysis. However, this meta-analysis is not perfect either, as ideally the cases and controls should originate from the same population and in our analysis we attempted to match on country of origin (see S4 Table) and this approach is statistically less powerful because of the uneven distribution of cases and controls, as might reflect the fact that the *PRNP* SNP p-value is also less significant in the meta-analysis than in the primary analysis. Still we acknowledge that the results are a call for caution and the association between *GRM8* genetic variants and sCJD should be confirmed in larger analysis with extensive control for population stratification.

*GRM8* encodes for mGluR8, a protein that belongs to the metabotropic glutamate receptor family, a family that has recently been linked to the transduction of physiological and cytotoxic signals mediated by PrP^C^. MGluR1 and mGluR5 have been shown to interact with PrP^C^, with such associations appearing important for promoting neurite outgrowth [9, 10]. Additionally, a recent study demonstrated that mGluR5 coupled with PrP^C^ mediated the cellular toxicity of soluble β-amyloid oligomers [10]. Further, in the APPswe/PS1dE9 Alzheimer’s disease mouse model, altered PrP^C^ processing and a selective increase in cortical mGluR1 expression has been reported. The authors hypothesized that complex processing of PrP^C^ in connection with mGluR1 over-expression is triggered by β-amyloid peptides [11]. In the setting of accumulation of PrP^Sc^, our immunohistochemical assessment of a limited sample of sCJD patients found that carriers of the risk allele at rs6951643 tended to have higher mGluR8 expression in microglial cells compared to non-carriers. Although our functional prediction analysis for rs6951643 was uninformative, it is of interest that a nearby variant in partial LD (rs34182595) is located in a micro-RNA target site and could influence gene expression.

In conclusion, our study has detected a *GRM8* genetic variant as a suggestive marker for sCJD risk mapping outside the *PRNP* region. Our findings provide evidence supporting a role for glutamate receptor signalling pathways in sCJD susceptibility. The involvement of glutamate receptor pathway in sCJD should be addressed in future studies in order to provide new insights in its pathogenesis. Our results underscore the importance of increasing sample sizes in future studies in order to augment the likelihood of detecting additional non-*PRNP* genetic risk factors for sCJD.

## Methods

### Ethics statement

The present study was conducted according to the revised Declaration of Helsinki and Good Clinical Practice guidelines. A signed informed consent to participate in genetic research was obtained from all participants or patients’ relatives. The study was approved by Comité de Ética de la Investigación y de Bienestar Animal (CEIyBA), National Health Institute Carlos III and Comité de Ética de Ensayos clínicos de Galicia and Comite de Ética de la Fundación Pública Gallega de Medicina Genómica (Servicio Gallego de Salud, SERGAS) (Spain); the Lothian Health Board, Lothian Research Ethics Committee (reference MCO/103/90) (UK); the Ethik-Kommission der Universitätsmedizin Göttingen (No. 9/6/0) (Germany); the Ethic Committee of the Istituto Superiore di Sanità (CE-ISS 09/266) (Italy); the Medical Ethics Committee of The Erasmus MC and the review board of The Netherlands Ministry of Health Welfare and Sports (The Netherlands); the Ethics Committee of the Medical University of Vienna (396/2011) (Austria); the Human Research Ethics Committee, based at The University of Melbourne (941450) (Australia).

### Populations and study design

All patients were ascertained by National CJD Surveillance Centers. Only definite or probable sCJD cases, according to accepted classification criteria, were included [12]. Table 5 summarizes the study design and populations included after quality control. All cases and controls were of Caucasian origin. Legal representatives gave written informed consent, and all samples were taken in accordance with the Helsinki declaration.

**Table 5:** Study Design and populations tested.

STAGES	POPULATION EFFECTIVELY ASSESSED*	
	sCJD CASES (N)	CONTROLS (N)
**Stage 1: Discovery**	Germany (113)	
Genotyping method: Affymetrix 500k array	UK (269)	UK WTCCC controls (1482)
Number of SNPs effectively assessed 279389	Netherlands (52)	Netherlands-RS controls (457)
Number of SNPs selected for replication: 23	**TOTAL sCJD (434)**	**TOTAL controls (1939)**
**Stage 2: Replication in independent sCJD Cases**	Germany (284)	
Genotyping method: Sequenom iPLEX GOLD	Netherlands (76)	
Number of SNPs effectively assessed: 22	Italy (292)	
Number of SNPs replicated: 5	Australia (48)	
	France (150)	
	Spain (203)	
	Austria (56)	
	**TOTAL sCJD (1109)**	
**Stage 3: Replication in independent Controls**		Spain-USC (2193)
Genotyping method: Sequenom iPLEX GOLD		Netherlands-RS (71)
Number of SNPs effectively assessed: 5		**TOTAL controls (2264)**
Number of SNPs replicated: 2		
		**TOTAL controls (4203)**
**POOLED ANALYSIS**	**TOTAL sCJD (1543)**
		Netherlands-RS (6192)
		British in England and Scotland-10KG (137)
**Controls added in analysis stratified by country**		Toscani in Italy-10KG (385)
		Iberian populations in Spain-10KG (217)
		Utah residents with Northern and Western European ancestry-10KG (290)
	**TOTAL sCJD (1543)**	**TOTAL controls (11424)**

*Number of samples effectively genotyped that passed quality control

sCJD, sporadic Creutzfeldt-Jakob disease; WTCCC, Welcome Trust Case Control Consortium; RS, Rotterdam Study; USC, University of Santiago de Compostela; 10KG, 1000 Genome Project.

### Genotyping and quality control

At the discovery stage (stage one) patient’s samples were characterized using the Affymetrix 500k array at the USC node of the Spanish National Genotyping Center. Out of the initially available 554 sCJD samples there was not enough DNA in 61 (36 samples from the UK and 25 from Germany), leaving 493 for genotyping. Genotypes were called first by the Dynamic Model (DM) and those samples with an overall call rate >93% were subsequently called by a Bayesian Robust linear model with Mahalanobis distance (BRLMM). Samples with BRLMM call rates <95% and with call and discrimination rates of the Modified Partitioning Around Medoids algorithm (MDR-MCR) >10% were excluded (31 patient samples out of 493 did not reach these thresholds, leaving in total 462 available for the analysis). Genotype and quality control of the WTCCC controls have been described elsewhere [13]. The other set of controls (Rotterdam Study) were genotyped at the Genetic Laboratory, Department of Internal Medicine at Erasmus Medical Center (Rotterdam) also using the Afymmetrix 500K array following same protocols as those used for cases. After quality control, 28 patients and 25 controls were excluded resulting in a final discovery population of 434 sCJD patients and 1,939 controls. Replication was attempted in independent sCJD patients (n = 1,201) (stage two) and control series (n = 2,264) (stage three) with the Sequenom iPLEx GOLD platform. SNPs for replication were selected as follows: after excluding outliers, we chose the 10 SNPs with lowest p-values (when a cluster of SNPs was in LD tagging the same gene we selected only the one with lowest p-value) and the remainder from the top 100 when a) they were in linkage disequilibrium with other top 100 SNPs or b) agreed in direction with the previous sCJD GWAS [6], or c) were connected to pathways of interest based on our previous study [14] (phosphatidylinositol) or based on the pathway analyses performed with the current data (glutamate receptors).

### Statistical analysis of genetic data

The statistical analyses were conducted using GenABEL [15]. For the individual SNP analysis, we excluded those SNPs: 1) with call rates <98% within either group (n = 219,182); 2) with minor allele frequency<0.01 (n = 1); 3) with controls not in Hardy-Weinberg equilibrium (False Discovery Rate for unacceptably high individual heterozygosity<1%) (n = 1,300). The quality control further included a check of the sex chromosomes against the reported sex and unexpected sample duplicates (Identity by State, IBS>95%). All quality control was performed with the ‘check.marker’ function of GenABEL. After quality control, 279,389 out of 499,872 SNPs were included in the analyses. In the discovery analyses (stage one), we conducted a one degree of freedom additive score test between cases and controls coding the presence of the minor genotype 0 for non-carriers, 1 for heterozygous and 2 for homozygous carriers. We controlled subpopulation structure using principal components analysis (PCA). In brief, we selected a random set of 10,000 SNPs and calculated a genomic kinship matrix using pair wise identity by state statistic (function 'ibs'). We then performed PCA analysis (function 'cmdscale'), and adjusted our analysis by the three main IBS matrix principal components (function 'mlreg'). We calculated the genomic inflation factor lambda (λ) for both autosomal and X chromosome SNPs. In the replication phase (stages two and three), we compared allele frequencies in cases and controls by a Chi-squared test implemented in Haploview [16]. We used a Bonferroni correction to adjust for multiple testing, setting the threshold for significance to a p-value of 0.0023 (0.05/22 SNPs successfully genotyped). We combined the gene discovery and replication series of patients for all validated SNPs and used as the criterion for genome wide significance a p-value of 5x10^-8^. We attempted to adjust for country of origin after stage three. We analysed the data from stage two and three with genotypes of the *1000 Genomes Project* [17] and imputed genotypes from the *Rotterdam Study* [18]. We extracted the SNPs from the *1000 Genomes* reference set excluding the children and other family members (Version: phase I v3). Additionally we imputed the same SNPs from the *Rotterdam Study* (imputations of the same phase I v3of the *1000 Genomes*). Imputed genotypes had a very high quality score (R^2^ > = 0.99). Next we paired our cases and controls by country of origin (S4 Table) and calculated effect estimates of the SNPs per country. The effect estimates of the SNPs from the discovery stage (adjusted by PCAs) and the effect estimates from the replication were meta-analysed using a inverse variance weighted meta-analysis(METAL version released 2011-03-25). [19]. We performed pathway analyses using ALIGATOR, a method for studying groups of genes by testing for over-representation of members of those groups within lists of genes containing significantly associated SNPs from GWA studies [20]. This analysis used 410 SNPs with a p-value for significance of <0.001 out of 115,565 within-gene SNPs from a total of 279,389 available. We found these SNPs were associated with 94 genes from a total of 13,092 genes with GO annotation covered in this study. Additionally we used Ingenuity Pathway Analysis (IPA) www.ingenuity.com) to determine the functional pathways in the genes tagged by our top ranked SNPs. We again selected those genes tagged by SNPs with p-value <0.001 and selected the canonical pathway analysis implemented in IPA software.

### Sequencing

We sequenced the 11 exons and intronic flanking areas of the *GRM8* gene in 96 sCJD patients using specific primers (S5 Table). The amplification reactions were carried out with 25 ng of genomic DNA and 0.5 units of Taq DNA Polymerase in a volume of 25 μl. The final concentrations of other reactants were: 1x Taq DNA Polymerase Buffer, 0.1 mM dNTPs and 0.1 μM of each primer. The final concentration of MgCl_2_ was 2 mM for the amplification of exons 1–7 and 10; 4 mM for exons 8 and 9a; 1.5 mM for exon 9b and 1 mM for exon 11. Additionally, the amplification reactions of exons 1 and 8 were supplied with DMSO 10% and 5%, respectively. The PCR cycling conditions were as follows: initial denaturation at 96°C for 3 min followed by 30 cycles of 96°C for 30 s, annealing temperature (see S5 Table) for 30 s and extension temperature of 72°C for 1 min with a final extension at 72°C for 10 min. A 2 μl aliquot of the amplification reaction was sequenced using 0.1μM of the above primers.

### Functional prediction analysis

Functional prediction analysis of genetic variants was performed by the use of FuncPred online software (http://snpinfo.niehs.nih.gov/snpinfo/snpfunc.htm) [21]. Exonic splicing enhancers were analyzed by RESCUE-ESE Web Server (http://genes.mit.edu/burgelab/rescue-ese/) [22] and PITA online software (http://genie.weizmann.ac.il/pubs/mir07/index.html) [23] was used to assess potential micro-RNA target sites. We searched for correlations between our genetic variants and eQTL in GTEX [24] and Genenetwork (http://genenetwork.nl:8080/GeneNetwork/) [25].

### Phenotypic correlations

We performed an immunohistochemical study in brain samples from 48 sCJD patients. The rs6951643 genotype distribution was as follows: 13 AA, 25 AG, and 9 GG. Sections from the hippocampal region CA1 sub-region, temporal cortex and white matter were immunostained for mGluR8. The intensity of mGluR8 (1:50; polyclonal rabbit antibody, Novus Biologicals, Cambridge, UK) immunostaining was evaluated using a scale of 0–3 (0: no; 1: weak; 2: moderate; 3: strong staining). The frequency of mGluR8 positive cells was scored semi-quantitatively using three categories: 1, <10%; 2, 10–50%; 3, >50% and was evaluated to provide information about the relative number of mGluR8 positive cells within the tissue. The product of these two values (intensity and frequency scores) was used to give the overall scores (total scores). In adjacent sections microglial activation using immunostaining for HLA-DP, DQ, DR (clone CR3/43, 1:00, monoclonal mouse), and spongiosis were also estimated semi-quantitatively. The analysis was performed (GGK) blinded to rs6951643 genotype. Ordinal regression was employed to test for association between rs6951643 genotypes and brain semi-quantitative expression of mGluR8. We adjusted by disease duration, *PRNP* M129V genotype, gliosis and spongiosis. We correlated clinical variables (age at death and patient’s disease onset) with the presence of 0, 1 or 2 sCJD risk alleles. In order to assess rs6951643 influence on age at onset and disease duration we performed a time to event analysis using Cox regression adjusting by *PRNP*M129V genotype and other relevant factors like sex and country of origin.

## Supporting Information

S1 Fig Q-Q plots for autosomal and X chromosome SNPs. (TIF)

S2 Fig Genotype clusters of the five SNPs studied in stage three with Sequenom iPLEx GOLD. (TIF)

S3 Fig Semi-quantitative assessments of mGluR8 expression in microglia (0 to 3) in the temporal region across the three rs6951643 genotypes in 48 sCJD patients. (TIF)

S1 Table Demographic and clinical features of cases. (DOCX)

S2 Table Top 100 SNPs after GWA analysis. (XLS)

S3 Table Pathway analysis with ALIGATOR. (XLS)

S4 Table Meta-analysis Sample population matching. (DOCX)

S5 Table Pairs of primers used for sequencing of the 11 exons and intronic flanking areas of GRM8 gene. (DOCX)

S6 Table GWA analysis results X chromosome SNPs. (ZIP)

S7 Table GWA analysis results autosomic SNPs. (ZIP)
