# Supplementary figures and images for: A Genome Wide Association Study Links Glutamate Receptor Pathway to Sporadic Creutzfeldt-Jakob Disease Risk
Source: PLoS One. 2015 Apr 28;10(4):e0123654. doi: 10.1371/journal.pone.0123654 (PMC4412535; doi:10.1371/journal.pone.0123654)

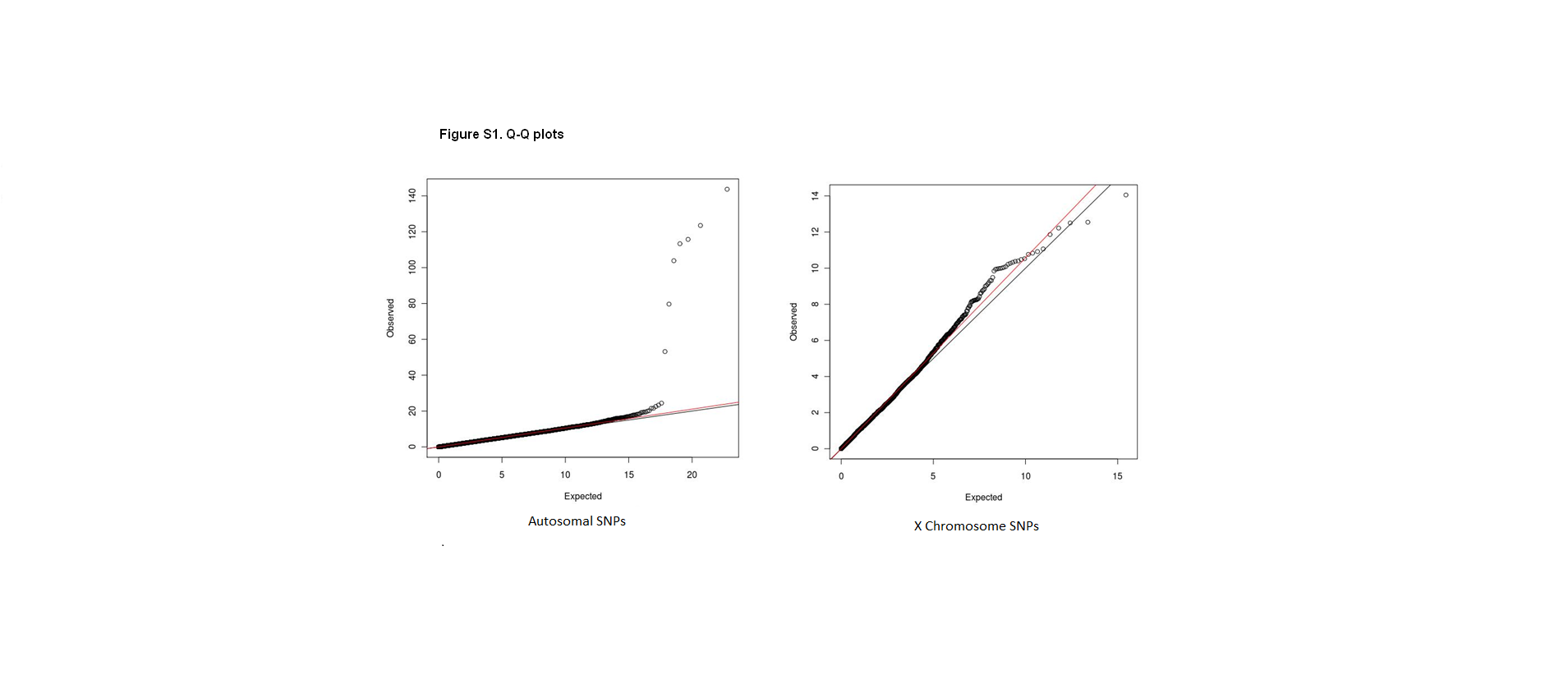

Supplement: S1 Fig — (TIF) [file pone.0123654.s001.tif]

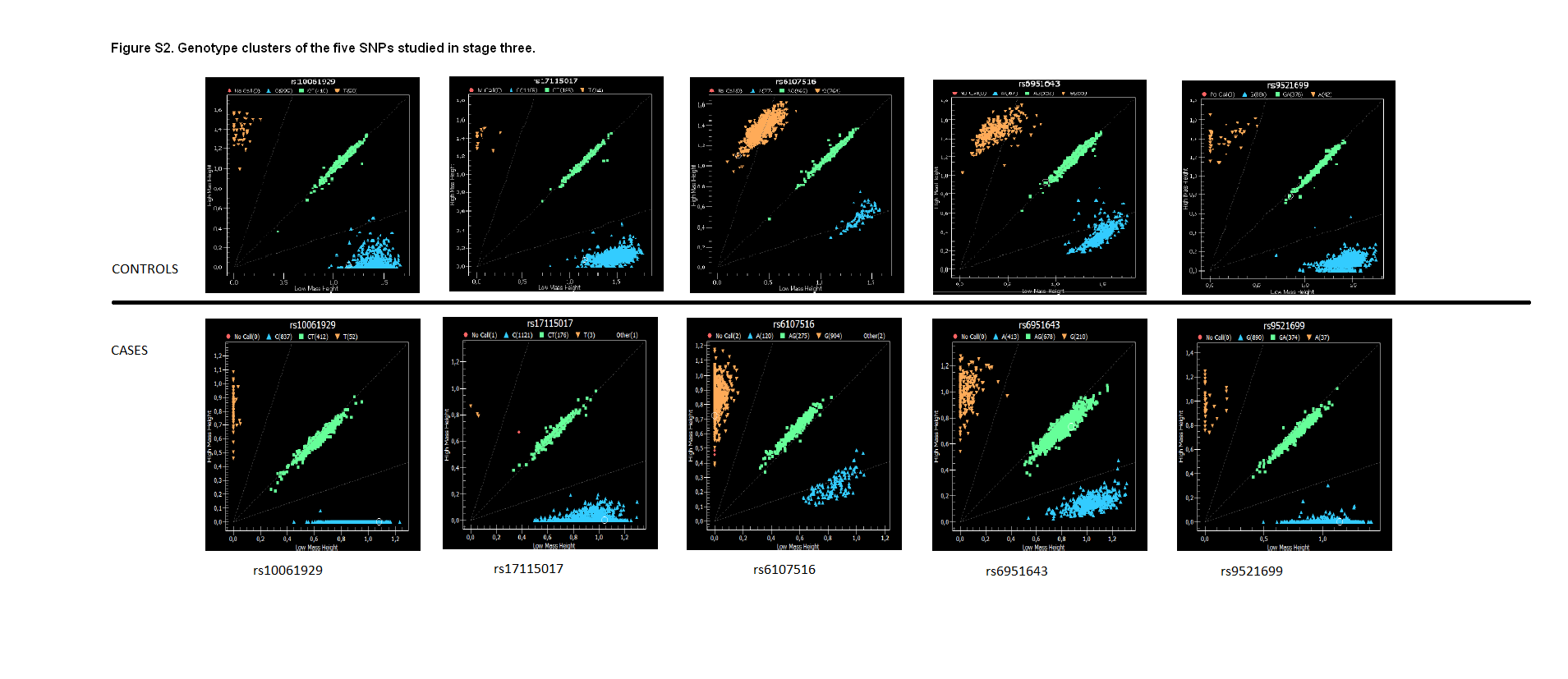

Supplement: S2 Fig — (TIF) [file pone.0123654.s002.TIF]

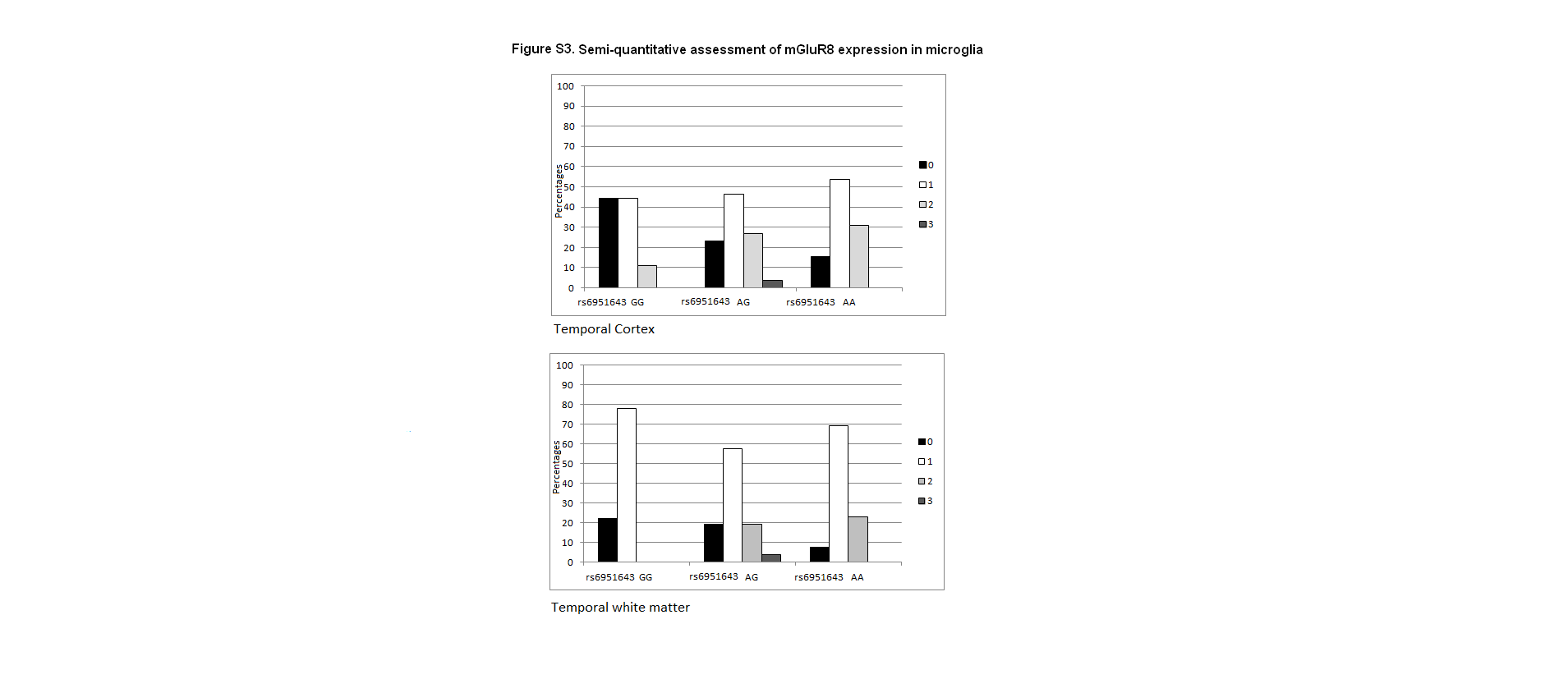

Supplement: S3 Fig — (TIF) [file pone.0123654.s003.tif]
