# Supplementary material for: A Genome Wide Association Study Links Glutamate Receptor Pathway to Sporadic Creutzfeldt-Jakob Disease Risk
Source: PLoS One. 2015 Apr 28;10(4):e0123654. doi: 10.1371/journal.pone.0123654 (PMC4412535; doi:10.1371/journal.pone.0123654)
Supplement: S1 Table — (DOCX) [file pone.0123654.s004.docx]

**Table S1. Demographic and clinical features of cases**

|  | Pooled population  (n=1543) | Discovery Population  (n=434) | Replication poulation  (n=1109) | P-values |
| --- | --- | --- | --- | --- |
|  |  |  |  |  |
| Definite diagnosis (%) | 62.5 | 77.1 | 59.3 | <0.001 |
| Females (%) | 52.6 | 53.7 | 54.3 | 0.83 |
| Median age at death (Range) | 66.9 (20-91) | 66.6 (20-88) | 67.1 (27-91) | 0.44 |
| Median disease duration in months (Range) | 7.4 (1-62) | 7.3 (1-54) | 7.4 (1-62) | 0.89 |
|  |  |  |  |  |
