# Supplementary material for: A Genome Wide Association Study Links Glutamate Receptor Pathway to Sporadic Creutzfeldt-Jakob Disease Risk
Source: PLoS One. 2015 Apr 28;10(4):e0123654. doi: 10.1371/journal.pone.0123654 (PMC4412535; doi:10.1371/journal.pone.0123654)
Supplement: S4 Table — (DOCX) [file pone.0123654.s007.docx]

| **Supplementary Table 4. Meta-analysis Sample population matching** | | | | |
| --- | --- | --- | --- | --- |
|  | | |  |  |
|  | **sCJD CASES** | **CONTROLS** | |  |
|  | Australia | British in England and Scotland-10KG | |  |
|  | Netherlands and Germany | Rotterdam study imputed genotypes | |  |
|  | Italy | Toscani in Italy-10KG | |  |
|  | Austria and France | Utah residents with Northern and Western European ancestry-10KG | |  |
|  | Spain | Spanish controls (stage 2 analysis) - Iberian populations in Spain-10KG | |  |

sCJD, sporadic Creutzfeldt-Jakob disease; RS, Rotterdam Study; USC, University of Santiago de Compostela; 10KG, 1000 Genome Project.
