# Supplementary material for: A Genome Wide Association Study Links Glutamate Receptor Pathway to Sporadic Creutzfeldt-Jakob Disease Risk
Source: PLoS One. 2015 Apr 28;10(4):e0123654. doi: 10.1371/journal.pone.0123654 (PMC4412535; doi:10.1371/journal.pone.0123654)
Supplement: S5 Table — (DOCX) [file pone.0123654.s008.docx]

**Table S5. Pairs of primers used for sequencing of the 11 exons and intronic flanking areas of *GRM8* gene.**

| **EXON (number)** | **T_m_**  **(ºC)** | **Forward** | **Reverse** |
| --- | --- | --- | --- |
| **1** | 57 | GTAGATGGCCCTGCATGTCC | CGTGCTAACACGGAGAACGC |
| **2** | 57 | GTTTAGATGCAGTGCAGCCAG | GAATGTCCCATTTGAGGAACC |
| **3** | 57 | GAGAAAGTAAGCCTATGGGCC | CAGTGGCTCTAATGTGCCAG |
| **4-5*** | 57 | CTGTCACTTGTCCTTTGGCTTATG | CCTTTGAGTTCAGATCCAACTGG |
| **6** | 57 | CACCAGGCTTTCCTGTTGTAC | GTATGCTCAGCCCTGAGACAC |
| **7** | 57 | GACACTCCTGGAGCTGAGACAG | CCACTCTGCCTGGGTATCTTC |
| **8** | 58 | GTTATGTGCATTGCTTAACTTGG | CATATTCAGTCGATTCCACACC |
| **9a**** | 63 | CTCCAGATGTCACATTGGTCAGC | GAACTTGGGCGCTGTGACAG |
| **9b**** | 65 | CTTCCGACGGGTCTTCCTAGG | CAATGTGACCTTGACGGATGG |
| **10** | 57 | GCAGGTTAGGACACAGGAATG | GTAGAACAGCCGCTCATCTTG |
| **11** | 57 | GCTCCATTATAACTCATGGGACC | CATCAGGGCTTTCATAGAAGGAG |

*Exons 4 and 5 were sequenced jointly in a single PCR, due to their proximity.

**Exon 9 was divided in two PCR reactions (9a and 9b) because of its large size.

Tm temperature of melting of each amplification reaction.
